# Accessible 2D video-based system for gait kinematic analysis: an inter-rater reliability study

**DOI:** 10.3389/fbioe.2026.1815411

**Published:** 2026-07-20

**Authors:** Albert Puig-Diví, Lluís Costa-Tutusaus, Juanjo García-Gil, Francesco Sartori, Javier Picañol

**Affiliations:** 1 Blanquerna School of Health Sciences, Universitat Ramon Llull, Barcelona, Spain; 2 Department of Health Sciences, TecnoCampus University Center, Pompeu Fabra University, Barcelona, Spain; 3 Neurophysiology Laboratory, Department of Biomedicine, Faculty of Medicine and Health Sciences, Institute of Neurosciences, University of Barcelona, Barcelona, Spain

**Keywords:** gait analysis, inter-rater reliability, kinovea, lower-limb kinematics, spatiotemporal parameters, two-dimensional analysis

## Abstract

**Introduction:**

Quantitative gait analysis supports clinical decision-making and outcome monitoring, yet its use remains largely restricted to specialized laboratories due to cost and operational complexity. Smartphone-based two-dimensional video approaches using open-source software have shown promising validity, but inter-rater reliability—particularly the influence of examiner expertise across spatiotemporal and kinematic outcomes—remains insufficiently characterized in powered samples.

**Methods:**

In this observational study, 84 healthy adults were included and recorded during level barefoot walking using a standardized smartphone setup. Four examiners (two experienced, two non-experienced) independently extracted spatiotemporal parameters and hip, knee, and ankle joint angles at different gait events using Kinovea. Inter-rater reliability was assessed with intraclass correlation coefficients (two-way mixed-effects, absolute agreement; ICC [3,1]) with 95% confidence intervals, and measurement error was quantified using the standard error of measurement (SEM) and minimal detectable change (MDC95). Agreement between expert and non-expert examiners was explored using Bland–Altman plots for representative variables. Mean values were descriptively compared with established three-dimensional reference values.

**Results:**

Global spatiotemporal descriptors demonstrated excellent reliability with minimal error and limited dependence on examiner expertise (walking speed ICC = 0.96; cadence ICC = 0.98; stride length ICC = 0.96). In contrast, temporal gait phase variables showed poor reliability overall (ICC ≈ 0.31–0.43), with higher—but still limited—agreement among experienced raters. Joint kinematics exhibited joint- and event-dependent reliability following a proximal-to-distal gradient: hip angles showed the highest consistency (ICC up to 0.88), knee angles moderate-to-good agreement (ICC ≈ 0.65–0.85), and ankle angles the greatest variability, particularly for event-dependent measures (e.g., opposite toe-off ICC = 0.45). Bland–Altman analyses revealed negligible bias for spatiotemporal outcomes but wider limits of agreement for kinematic variables. Mean values were biomechanically coherent, although systematic differences in joint angles were observed when compared with 3D reference data.

**Conclusion:**

In healthy young adults, a smartphone- and Kinovea-based workflow can provide reliable spatiotemporal gait metrics and moderate-to-good reliability for selected proximal kinematics, whereas temporal gait phase variables and distal joint angles remain less reliable and should be interpreted cautiously in longitudinal or multi-examiner contexts.

## 1 Introduction

Human gait is a fundamental functional behavior and an indicator of neuromusculoskeletal integrity. Quantitative gait assessment plays a role in research as well as clinical decision-making, rehabilitation, injury prevention, and performance monitoring across neurological, musculoskeletal, and aging-related conditions (Bonacina et al., 2024; Chen et al., 2024; Faj et al., 2025; Nourbakhsh et al., 2025). Since early works, the conceptual framework of the gait cycle has provided a standardized reference for describing spatiotemporal and kinematic events, enabling comparability across studies and clinical contexts (Perry and Burnfield, 2010). Despite this, quantitative gait analysis remains inconsistently implemented in routine practice.

Three-dimensional (3D) motion capture systems remain the reference standard for kinematic gait analysis (Scataglini et al., 2024). These laboratory-based platforms provide high-resolution spatial and temporal data. However, they require specialized infrastructure, technical expertise, and substantial financial investment for both acquisition and maintenance (Thewlis et al., 2013; Scataglini et al., 2024). Their operation entails considerable time demands for setup, calibration, data processing, and analysis. As a result, despite their methodological robustness, 3D laboratories are not routinely accessible in many clinical environments. This structural limitation has created a tension between the recognized value of quantitative gait assessment and its practical feasibility in real-world settings. Consequently, there has been growing interest in scalable motion analysis solutions that aim to preserve clinically meaningful quantitative information while reducing barriers (Springer and Yogev Seligmann, 2016; Spanos et al., 2023). Early attempts at simplified gait analysis systems emerged more than a decade ago (Soda et al., 2009), with subsequent portable approaches demonstrating feasibility for basic spatiotemporal variables—including stride length, cadence, stance duration, and walking speed—in small validation samples (Macleod et al., 2014). Together, these developments have laid the groundwork for broader exploration of accessible gait analysis methodologies.

In recent years, markerless motion capture systems based on computer vision and deep learning have emerged as an alternative to both laboratory-based and simplified video analysis approaches. These systems, including widely used frameworks such as OpenPose, MediaPipe, and DeepLabCut, enable automated extraction of kinematic data from two-dimensional or multi-view video without the need for reflective markers. Their scalability and minimal hardware requirements have facilitated movement analysis in non-laboratory environments. Validation studies report promising agreement with marker-based motion capture systems for selected kinematic and spatiotemporal variables, although accuracy remains dependent on task complexity, camera configuration, and algorithm performance, particularly in the presence of occlusions or out-of-plane motion (Cheng et al., 2025; Unsihuay et al., 2026; Verheul et al., 2026). In addition, variability across algorithms, limited standardization, and challenges in clinical interpretability remain relevant limitations. In this context, semi-automated video-based approaches such as Kinovea remain relevant due to their accessibility and user-guided analysis. Rather than aiming for full automation, these approaches allow direct control over landmark identification and measurement, positioning them as an intermediate solution between observational gait analysis and fully automated markerless systems.

Kinovea has emerged as one of the most widely adopted open-source platforms for two-dimensional motion analysis. Initial validation studies supported its accuracy for time-based variables through comparison with force-platform-derived measurements (Balsalobre-Fernández et al., 2014), and subsequent work extended validation to angular and distance parameters (Puig-Diví et al., 2019). Later investigations compared Kinovea-derived kinematics with 3D motion capture systems, reporting acceptable agreement for selected joint angles and spatiotemporal descriptors under controlled experimental conditions (Fernández-González et al., 2020; Hunter et al., 2022; Iijima et al., 2023; Menychtas et al., 2023; Faj et al., 2025; Vicente-Pina et al., 2025). Beyond methodological validation, Kinovea has been applied in diverse clinical and performance contexts, including gait assessment in neurological populations, musculoskeletal injury analysis, and sport-specific biomechanical evaluation (Marotta et al., 2020; Poncela-Skupien et al., 2020; Nitayarak and Charntaraviroj, 2021; Souissi et al., 2021; Rashid et al., 2023; Yodchaisarn et al., 2023; Zarei et al., 2023; Alsakhawi et al., 2024; Kaya Aytutuldu et al., 2024; Lashien et al., 2024; Martins et al., 2024; Kumari et al., 2025; Utku Umut et al., 2025; Shabana et al., 2026). In some instances, it has also served as a reference method in studies evaluating emerging motion analysis tools or digital health applications (Sabino et al., 2021; Ceballos-Laita et al., 2023; Ciucurel and Iconaru, 2024; Langley et al., 2025; Lauman et al., 2025; Martins et al., 2025; Odabaşıoğlu et al., 2025; Sharan et al., 2025; Soylu et al., 2025; Suansomchit et al., 2025; Ziaiee et al., 2025).

Although Kinovea has been validated for time-based, angular, and distance measurements under controlled conditions (Balsalobre-Fernández et al., 2014; Puig-Diví et al., 2019), and several studies have reported acceptable agreement for selected variables (Fernández-González et al., 2020; Hunter et al., 2022; Iijima et al., 2023; Menychtas et al., 2023; Faj et al., 2025; Vicente-Pina et al., 2025), evidence specific to comprehensive gait analysis remains limited and methodologically fragmented. Existing investigations have typically focused either on joint angles at isolated gait events or on selected spatiotemporal descriptors, rarely integrating both domains within a full-cycle analytical framework (Fernández-González et al., 2020; Spanos et al., 2023). Moreover, most studies have emphasized concurrent validity relative to laboratory systems, whereas examiner-related variability and measurement error—critical determinants of clinical applicability—have received comparatively little attention.

Consequently, it remains unclear to what extent a smartphone-based two-dimensional workflow can provide internally consistent spatiotemporal and kinematic measurements across multiple gait events. Beyond agreement with three-dimensional reference values, establishing inter-rater reliability and quantifying measurement error are necessary to determine which variables are suitable for longitudinal monitoring and multi-examiner implementation in healthy young adults. Accordingly, this study aimed to evaluate the inter-rater reliability of a smartphone-based, sagittal-plane two-dimensional gait analysis workflow using Kinovea in healthy adults. We quantified agreement and measurement error for spatiotemporal parameters and lower-limb joint angles across predefined gait events and examined the influence of examiner expertise.

## 2 Methods

### 2.1 Study design

An observational study was conducted to evaluate the inter-examiner reliability of spatiotemporal and kinematic gait parameters derived from smartphone-recorded videos and analyzed using Kinovea software. The study was designed and reported in accordance with the Guidelines for Reporting Reliability and Agreement Studies (GRRAS) (Kottner et al., 2011). Eighty-four participants were independently assessed by four examiners (two experienced and two non-experienced) to evaluate inter-examiner reliability overall and within examiner subgroups.

### 2.2 Participants

Eighty-four healthy young adult participants were recruited using a convenience sampling strategy. Gait recordings were obtained from all participants under standardized conditions and included in the present study. Participants were eligible for inclusion if they met all of the following criteria: (i) male or female adults aged 20–60 years; (ii) independent ambulation without assistive devices; (iii) ability to perform continuous level walking; and (iv) ability to follow instructions and complete the assessment protocol. Participants were excluded if any of the following were present: (i) history or presence of central neurological disorders (e.g., stroke, multiple sclerosis, Parkinson’s disease, traumatic brain injury with sequelae); (ii) chronic musculoskeletal pathology or dysfunction affecting gait (lower limb or trunk), including osteoarthritis, chronic tendinopathy, or recurrent instability; (iii) lower-limb surgery, or acute musculoskeletal injury of the lower limbs within the previous year; (iv) moderate to severe visual impairment not adequately corrected, or moderate to severe vestibular dysfunction; (v) musculoskeletal pain at the time of assessment; (vi) use of medications or substances with relevant acute effects on balance, coordination, or gait (e.g., sedatives, alcohol) within 24 h prior to testing; and (vii) any other condition deemed by the investigators to compromise safe participation or to materially affect gait biomechanics. The study was approved by the Ethics Committee of Ramon Llull University (CER URL; code URL_2022_2023_001, *“Kinematic parameters of normal human gait using a low-cost technology*,*”* Barcelona, Spain). All procedures were conducted in accordance with the ethical standards of the institutional research committee and with the Declaration of Helsinki. All participants provided written informed consent prior to participation, authorizing the use of their gait recordings for research purposes.

### 2.3 Procedure

Prior to gait recording, a baseline assessment was conducted immediately before data acquisition. Baseline variables included sex, age, body height, and body mass, from which body mass index (BMI) was calculated. Baseline assessments and gait recordings were performed during the same session, under controlled conditions. Each participant’s gait was recorded in the sagittal plane using a smartphone (Samsung Galaxy S21 Ultra, Samsung Electronics, Seoul, South Korea) at a frame rate of 60 frames per second (fps), which has previously been shown to provide adequate temporal resolution for spatiotemporal and kinematic gait analysis using Kinovea software. This recording setup, including frame rate and Full HD resolution (1920 × 1080 pixels), is consistent with prior validation and reliability studies employing similar smartphone-based methodologies (Spanos et al., 2023; Vicente-Pina et al., 2025).

**FIGURE 1:**
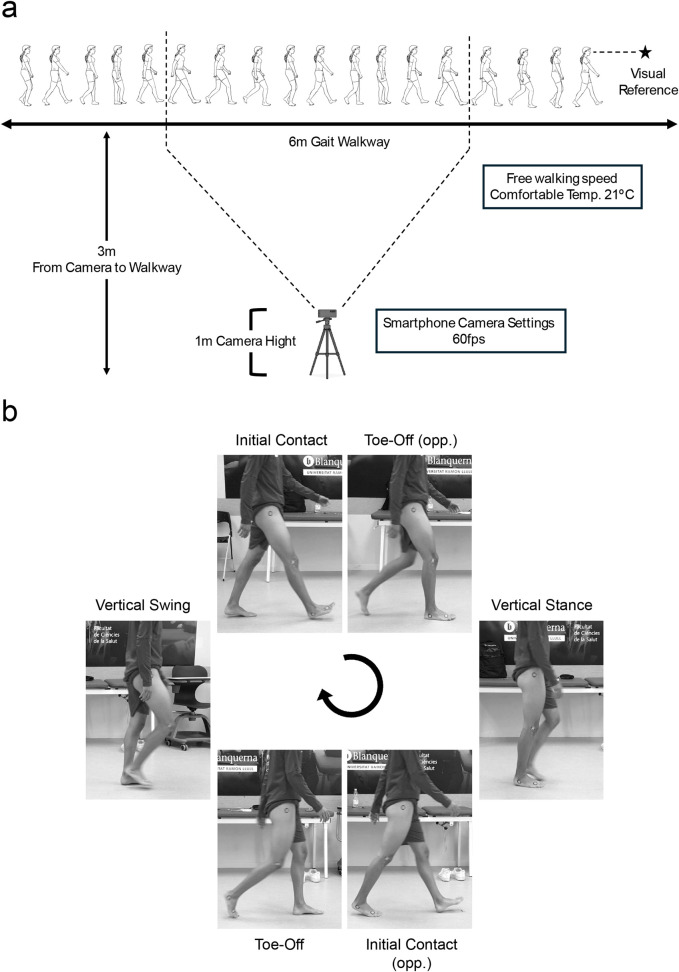
Experimental setup and gait event definition. **(a)** Schematic representation of the experimental setup used for gait recording. Participants walked barefoot at a self-selected comfortable speed along a 6 m gait walkway on a flat, rigid surface under consistent indoor lighting and comfortable environment conditions (21 °C). A visual reference positioned at eye level was used to standardize gaze direction during walking. Gait was recorded in the sagittal plane using a smartphone camera configured at 60 frames per second, mounted horizontally on a tripod placed 3 m from the walkway and 1 m above ground level. **(b)** Representative sagittal-plane video frames illustrating the key gait events used for analysis, with reflective markers visible at the anatomical landmarks (fourth metatarsal head, lateral calcaneus, lateral femoral epicondyle, and greater trochanter). The following gait events are shown in clockwise order: initial contact (IC), toe-off of the opposite limb (TOop), vertical stance (VSt), toe-off (TO), initial contact of the opposite limb (ICop), and vertical swing (VSw). These events were used to define the gait cycle and to extract spatiotemporal kinematic and angular parameters.

The smartphone was positioned horizontally on a tripod at 3 m from the walking pathway and at a height of 1 m from the ground (Figure 1a). Recordings were performed along a 6 m walking pathway on a flat, rigid surface, free of obstacles, under consistent indoor lighting and comfortable ambient conditions. For spatial calibration, two metric reference markers separated by 2 m were placed along the calibrated central region of the walking pathway. Participants were instructed to walk straight through this calibration zone, directly over the reference markers. This procedure ensured that the distance between the participant and the camera matched the distance between the calibration markers and the camera, thereby minimizing perspective-related scaling errors and ensuring accurate conversion from pixel-based measurements to real-world distances.

Participants were instructed to walk barefoot at a self-selected comfortable speed while maintaining their gaze on a visual reference positioned at eye level in front of the walkway, simulating natural overground walking. A 3-min familiarization period of free walking was completed prior to data acquisition, during which no gait cycles were recorded. When multiple gait cycles were visible, a single representative gait cycle was selected for analysis. The selected cycle corresponded to the stride in which the participant was best centered within the calibrated field of view and the analyzed limb was most orthogonal to the camera. This decision was made to standardize the analysis across participants and raters, minimize perspective-related errors in the two-dimensional sagittal-plane recording, and ensure that all examiners analyzed the same optimally visible cycle. Although averaging multiple gait cycles may provide a more robust estimate of an individual’s habitual gait pattern, the primary aim of the present study was to assess inter-rater reliability of a video-based measurement workflow under controlled conditions, rather than to characterize within-subject gait variability.

Four reflective markers (25 mm diameter), secured with hypoallergenic adhesive tape, were placed on predefined anatomical landmarks of one lower limb: the head of the fourth metatarsal, the lateral aspect of the calcaneus (below the lateral malleolus), the lateral femoral epicondyle, and the greater trochanter of the femur (Figure 1b). Marker size was selected based on previous studies for two-dimensional gait analysis (Iijima et al., 2023). Marker placement followed a standardized protocol adapted from previous methodologies, with minor modifications to accommodate the sagittal-plane recording approach used in the present study (Faj et al., 2025). To minimize variability, all markers were placed by the same trained examiner with prior experience in anatomical landmark identification. Landmarks were identified by palpation with participants standing barefoot in a neutral upright position, with weight evenly distributed. The fourth metatarsal head was identified at the dorsum of the forefoot, the calcaneal marker was positioned just inferior to the lateral malleolus, the lateral femoral epicondyle was located through gentle knee flexion–extension to identify the joint axis, and the greater trochanter was identified during passive hip rotation. Prior to recording, marker visibility was verified in the sagittal plane, and minor adjustments were performed when necessary to ensure consistent visualization and minimize occlusion throughout the gait cycle.

Reflective markers were used to improve landmark identification during examiner-based analysis and to enhance measurement consistency within the two-dimensional framework. While fully automated markerless systems based on computer vision and deep learning offer increased ecological validity and reduced operator interaction, they remain subject to variability related to algorithm performance, occlusions, and environmental conditions. In contrast, the marker-assisted approach employed in this study represents a controlled intermediate methodology, aimed at reducing landmark detection error and improving measurement repeatability, particularly in the context of inter-examiner reliability assessment. This approach allows the isolation of examiner-related variability from algorithm-dependent variability, thereby providing a more controlled framework to evaluate measurement consistency in a smartphone-based setting.

Subsequently, four examiners independently analyzed all gait recordings. The examiner panel consisted of four raters and was predefined based on prior experience in gait analysis. Experienced examiners were defined as senior faculty members in biomechanics with regular use of Kinovea for research purposes over the previous 5 years and prior experience in spatiotemporal and kinematic gait assessment. Non-experienced examiners were defined as junior researchers and/or clinicians without previous formal experience in gait analysis. All gait recordings were anonymized and assigned a coded identifier prior to analysis. Examiners were blinded to each other’s measurements, participant identity, and reference values of spatiotemporal and kinematic gait parameters obtained from the three-dimensional motion capture system.

### 2.4 Data processing

All gait recordings were independently analyzed by each examiner using Kinovea software (version 2023.1.2; open source), following a standardized written data processing protocol (Supplementary Material S1). Key gait events were defined according to established biomechanical criteria, based on previously published gait phase definitions, with minor adaptations to enhance clinical relevance in a two-dimensional sagittal-plane context (Perry and Burnfield, 2010).

Prior to formal data extraction, all examiners received the same written protocol, including operational definitions of each gait event, frame selection criteria, and instructions for spatial calibration and joint angle measurement. A familiarization session was conducted using practice videos not included in the final dataset, during which the analysis workflow was reviewed and potential ambiguities in event identification were discussed. No consensus correction was performed on the study recordings, and examiners remained blind to each other’s measurements throughout the analysis. This approach was chosen to ensure standardized application of the protocol while preserving independent ratings for inter-examiner reliability assessment. Although event identification was performed manually on a frame-by-frame basis, predefined criteria were applied to reduce variability in the selection of key gait events. Nevertheless, minor discrepancies in frame selection may occur, particularly for events such as toe-off and contralateral toe-off, where visual determination of ground contact may be less distinct in sagittal-plane recordings.

After this initial familiarization, key gait events were identified frame by frame in the sagittal plane (Figure 1b). The following events were defined: first initial contact (IC1), toe-off of the contralateral limb (TOop), vertical stance (VSt), initial contact of the contralateral limb (ICop), toe-off (TO), vertical swing (VSw), and second initial contact (IC2). When uncertainty existed regarding the exact frame corresponding to initial contact, the frame immediately following the last non-contact frame was selected to ensure consistent event identification (specific criteria available in Supplementary Material S2). Second, spatial calibration was performed using the two metric reference markers placed 2 m apart along the walking pathway, allowing the conversion of pixel-based measurements into real-world distances. Temporal variables were then obtained by chronometring the duration of the gait cycle (IC1–IC2) and its constituent phases, including stance, swing, single support, and double support. Based on these measurements, spatiotemporal gait parameters were calculated, including stride length, gait velocity, cadence, and the relative duration of stance, swing, single support, and double support phases, expressed as percentages of the gait cycle. Calculations were performed according to predefined formulas to ensure consistency across examiners (Supplementary Material S3).

Joint angles of the ankle, knee, and hip were extracted at each predefined gait event. Angles obtained directly from Kinovea were referenced to anatomical position to derive physiologically meaningful joint angles. Specifically, ankle and hip angles were calculated as 90° minus the angle provided by Kinovea, whereas knee angles were calculated as 180° minus the Kinovea-derived angle. Importantly, during specific gait phases (vertical stance and vertical swing), partial occlusion of the greater trochanter marker occasionally occurred due to overlapping with the upper limb in the sagittal plane. In such cases, marker position was inferred based on the visible trajectory before and after occlusion. This limitation is inherent to two-dimensional gait analysis and has been previously described in comparable 2D motion capture studies (Menychtas et al., 2023).

### 2.5 Statistical analysis

Statistical analyses were performed using RStudio (R Foundation for Statistical Computing, Vienna, Austria). All data were recorded and organized in independent databases using Microsoft Excel (Microsoft Corp., Redmond, WA, United States). Data distribution was assessed using the Shapiro–Wilk test and complemented by visual inspection of Q–Q plots. Descriptive statistics were calculated for all variables and are presented as mean values with measures of variability. Variability is reported as standard deviation (SD) or standard error of the mean (SEM), as appropriate. All estimates are reported with 95% confidence intervals (CI) unless otherwise stated. Inter-examiner reliability was assessed using intraclass correlation coefficients (ICC). ICCs were calculated in R using the *irr* package, applying a two-way mixed-effects model for absolute agreement and single measurements (ICC [3,1]). This model was selected because the same predefined examiners assessed all participants, examiner effects were therefore treated as fixed, and the objective was to quantify absolute agreement for individual measurements rather than consistency or generalizability to a broader population of raters. ICC values are reported together with their 95% confidence intervals. The magnitude of reliability was interpreted according to the thresholds previously proposed: values < 0.50 were considered poor, 0.50–0.75 moderate, 0.75–0.90 good, and >0.90 excellent reliability (Koo and Li, 2016). Reliability analyses were performed considering: (i) all four examiners jointly, and (ii) two examiner subgroups, defined *a priori* as experienced examiners and non-experienced examiners. Measurement error was quantified by calculating the standard error of measurement (SEM), derived from the pooled standard deviation and ICC values. The minimal detectable change at the 95% confidence level (MDC_95_) was subsequently calculated to estimate the smallest change exceeding measurement error. SEM and MDC_95_ were additionally computed for the average of examiners to reflect the reliability of mean values across raters. Agreement between examiners was further explored using Bland–Altman plots. These plots were constructed using the means of paired measurements plotted against their difference, allowing visual assessment of systematic bias and limits of agreement. Mean bias and 95% limits of agreement were calculated for each comparison. Intra-rater reliability was not calculated because each examiner analyzed each recording once. Data visualization and preliminary analyses were performed using GraphPad Prism (GraphPad Software, San Diego, CA, United States). Final figures were prepared using Adobe Illustrator (Adobe Inc., San Jose, CA, United States).

### 2.6 Sample size

Four examiners participated in the reliability analysis. This number was considered sufficient to evaluate overall inter-examiner agreement, to estimate reliability for averaged ratings, and to perform predefined subgroup analyses between experienced and non-experienced examiners, using a two-way ICC model with all examiners rating all participants. Sample size was determined *a priori* using a confidence-interval precision approach for ICC estimation. The calculation followed the precision-based approach proposed by Bonett (2002), using a closed-form analytical approximation for estimating the sample size required to achieve a desired confidence interval width for the ICC (Bonett, 2002). Assuming four raters (k = 4), an expected ICC of 0.80, a two-sided 95% confidence level, and a target confidence interval half-width of 0.08, the required sample size was estimated at 71 participants. The expected ICC of 0.80 was selected as a conservative threshold corresponding to good reliability, and the half-width of 0.08 was chosen to provide sufficiently precise ICC estimates while maintaining feasibility. Calculations were performed in R using a custom script. To account for potential data loss due to technical issues, unusable recordings, or protocol deviations, the planned sample size was increased by approximately 20%; therefore, 84 participants were included in the final analysis.

## 3 Results

### 3.1 Participant characteristics

A total of 84 healthy young adults were included in the study, comprising 34 females (40.5%) and 50 males (59.5%) (Table 1). The mean age of the overall sample was 21.56 ± 5.20 years. Mean height, body weight, and body mass index (BMI) were 172.66 ± 10.07 cm, 67.97 ± 12.98 kg, and 22.61 ± 2.63 kg/m^2^, respectively. The examiner panel consisted of four raters: two experts and two non-experts (Table 2). Expert examiners had a mean age of 54 ± 1.41 years and 30 ± 4.24 years since graduation. Both were male, held PhD degrees, had substantial teaching experience (19.5 ± 4.95 years), and prior experience using Kinovea (16.5 ± 3.54 years). Both held full professor positions at the time of the study. Non-expert examiners had a mean age of 27 ± 1.39 years and 6.5 ± 0.7 years since graduation. Both were male, held MSc degrees, had no formal teaching experience, and no prior experience using Kinovea. At the time of the study, they were enrolled as MSc students.

**TABLE 1:** Demographic and anthropometric characteristics of the study participants.

Characteristic	All	Female	Male
n, %	84, 100%	34, 40.48%	50, 59.52%
Age (years), mean ± SD	21.56 ± 5.20	21.71 ± 4.57	21.46 ± 5.63
Height (cm), mean ± SD	172.66 ± 10.07	163.67 ± 5.66	178.78 ± 7.49
Weight (kg), mean ± SD	67.97 ± 12.98	58.71 ± 10.31	74.28 ± 10.67
BMI (kg/m^2^), mean ± SD	22.61 ± 2.63	21.80 ± 2.95	23.20 ± 2.26

Abbreviations: SD, standard deviation; BMI, body mass index.

**TABLE 2:** Demographic and professional characteristics of the examiners.

Characteristic	Expert	Non-expert
Age (years), mean ± SD	54 ± 1.41	27 ± 1.39
Sex (male), n (%)	2 (100%)	2 (100%)
Academic degree, n (%)	PhD, 2 (100%)	MSc, 2 (100%)
Professional background	Physiotherapy and sport sciences	Physiotherapy
Years since graduation, mean ± SD	30 ± 4.24	6.5 ± 0.7
Teaching experience (years)	19.5 ± 4.95	0
Experience using kinovea (years)	16.5 ± 3.54	0
Current position, n (%)	Full professor, 2 (100%)	MSc students, 2 (100%)

Abbreviations: SD, standard deviation; MSc, Master of Science; PhD, doctor of philosophy.

### 3.2 Inter-examiner reliability of spatiotemporal gait parameters

**FIGURE 2:**
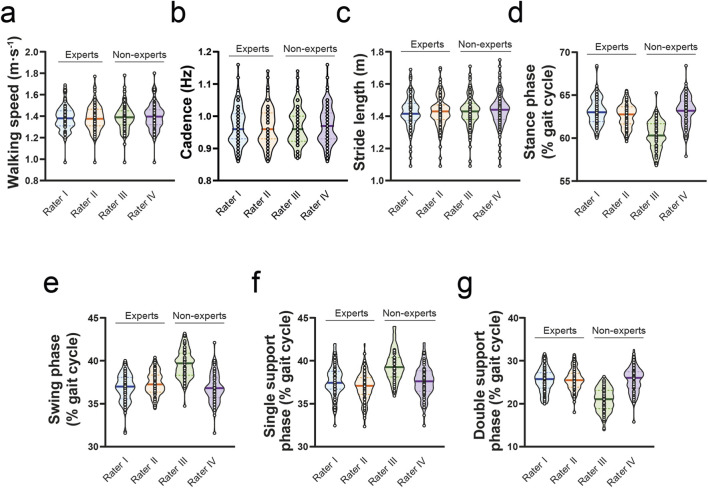
Video-derived spatiotemporal gait parameters across examiners. **(a)** Walking speed (m·s^-1^) measured independently by four raters, showing comparable central tendencies across expert (Raters I–II) and non-expert raters (Raters III–IV) in n = 84 participants. **(b)** Cadence (Hz), with narrow dispersion and substantial overlap between expert and non-expert measurements. **(c)** Stride length (m) obtained after spatial calibration, displaying similar distribution shapes across raters and minimal between-rater spread. **(d)** Stance phase duration (% gait cycle), exhibiting greater dispersion than speed-derived parameters, particularly among non-expert raters. **(e)** Swing phase duration (% gait cycle), showing complementary variability patterns to stance phase across raters. **(f)** Single-support phase (% gait cycle), with moderate between-rater variability and partially overlapping distributions. **(g)** Double-support phase (% gait cycle), displaying the largest dispersion among temporal gait phase variables, consistent with increased sensitivity to event detection across raters. For all panels, violin width reflects kernel density, horizontal lines indicate median values, and overlaid points represent individual participant measurements.

After gait recordings were obtained from the 84 participants, four independent raters analyzed spatiotemporal gait parameters using Kinovea software. Analyses were based on the identification of the seven gait events described in the Methods section, using three synchronized timers. Descriptive analysis using violin plots revealed broadly comparable distributions across raters for most spatiotemporal variables, including walking speed, cadence, stride length, stance phase, swing phase, single-support phase, and double-support phase (Figures 2a–g). However, visual agreement among the four raters was more consistent for walking speed, cadence, and stride length (Figures 2a–c). In contrast, greater variability was observed for temporal gait phase variables, with non-expert raters tending to diverge more noticeably from expert raters, particularly for stance phase, swing phase, and double-support phase estimates (Figures 2d–g).

Inter-examiner reliability outcomes for all spatiotemporal gait parameters, including intraclass correlation coefficients (ICC), 95% confidence intervals (95% CI), standard error of measurement (SEM), and minimal detectable change at the 95% confidence level (MDC95), are summarized in Table 3. Walking speed demonstrated excellent inter-examiner reliability, with an ICC of 0.96 (95% CI: 0.94–0.97), a SEM of 0.01 m s^-1^, and an MDC95 of 0.03 m s^-1^. Cadence showed similarly high agreement (ICC = 0.98, 95% CI: 0.97–0.98), with a SEM of 0.005 Hz and an MDC95 of 0.01 Hz. Stride length also exhibited excellent reliability, with an ICC of 0.96 (95% CI: 0.95–0.98), a SEM of 0.01 m, and an MDC95 of 0.03 m. These high ICC values were consistently observed in both expert (ICC range: 0.97–0.98) and non-expert raters (ICC range: 0.94–0.98), with experts showing smaller SEM and MDC95 values overall (Table 3).

**TABLE 3:** Inter-examiner reliability of spatiotemporal gait parameters for all examiners, experts, and non-experts.

Parameter	All				Experts				Non-experts			
	ICC	95% CI	SEM	MDC95	ICC	95% CI	SEM	MDC95	ICC	95% CI	SEM	MDC95
Stride length (m)	0.96	0.95–0.98	0.01	0.03	0.98	0.96–0.99	0.01	0.03	0.95	0.92–0.97	0.02	0.05
Walking speed (m·s^-1^)	0.96	0.94–0.97	0.01	0.03	0.98	0.97–0.99	0.01	0.03	0.94	0.91–0.96	0.02	0.06
Cadence (Hz)	0.98	0.97–0.98	0.005	0.01	0.97	0.95–0.98	0.01	0.02	0.98	0.96–0.98	0.006	0.02
Stance phase (%)	0.31	0.09–0.51	0.81	2.24	0.44	0.24–0.60	0.80	2.21	0.17	−0.09–0.42	1.40	3.87
Swing phase (%)	0.31	0.09–0.51	0.81	2.24	0.44	0.24–0.60	0.80	2.21	0.17	−0.09–0.42	1.40	3.87
Single-support phase (%)	0.43	0.21–0.61	0.75	2.09	0.53	0.33–0.67	0.87	2.42	0.31	−0.05–0.57	1.18	3.28
Double-support phase (%)	0.33	0.09–0.54	1.40	3.87	0.53	0.35–0.67	1.29	3.57	0.18	−0.09–0.44	2.35	6.52

Abbreviations: ICC, intraclass correlation coefficient; 95% CI, 95% confidence interval; SEM, standard error of measurement; MDC95, minimal detectable change at the 95% confidence level.

In contrast, temporal gait phase variables demonstrated poor inter-examiner reliability. Stance phase showed an ICC of 0.31 (95% CI: 0.09–0.51), with a SEM of 0.81% and an MDC95 of 2.24%. Swing phase presented comparable reliability (ICC = 0.31, 95% CI: 0.09–0.51), with a SEM of 0.81% and an MDC95 of 2.24%. Single-support phase exhibited slightly higher agreement but remained within the poor reliability range (ICC = 0.43, 95% CI: 0.21–0.61), with a SEM of 0.75% and an MDC95 of 2.09%. Double-support phase showed an ICC of 0.33 (95% CI: 0.09–0.54), with a SEM of 1.40% and an MDC95 of 3.87%. When stratified by examiner expertise, expert raters consistently demonstrated higher reliability for temporal gait phase variables (ICC range: 0.44–0.53, SEM range: 0.80%–1.29%, MDC95 range: 2.21%–3.57%) compared with non-expert raters (ICC range: 0.17–0.31, SEM range: 1.18%–2.35%, MDC95 range: 3.28%–6.52%), although reliability remained limited in both subgroups (Table 3).

### 3.3 Inter-examiner reliability of joint kinematics

**FIGURE 3:**
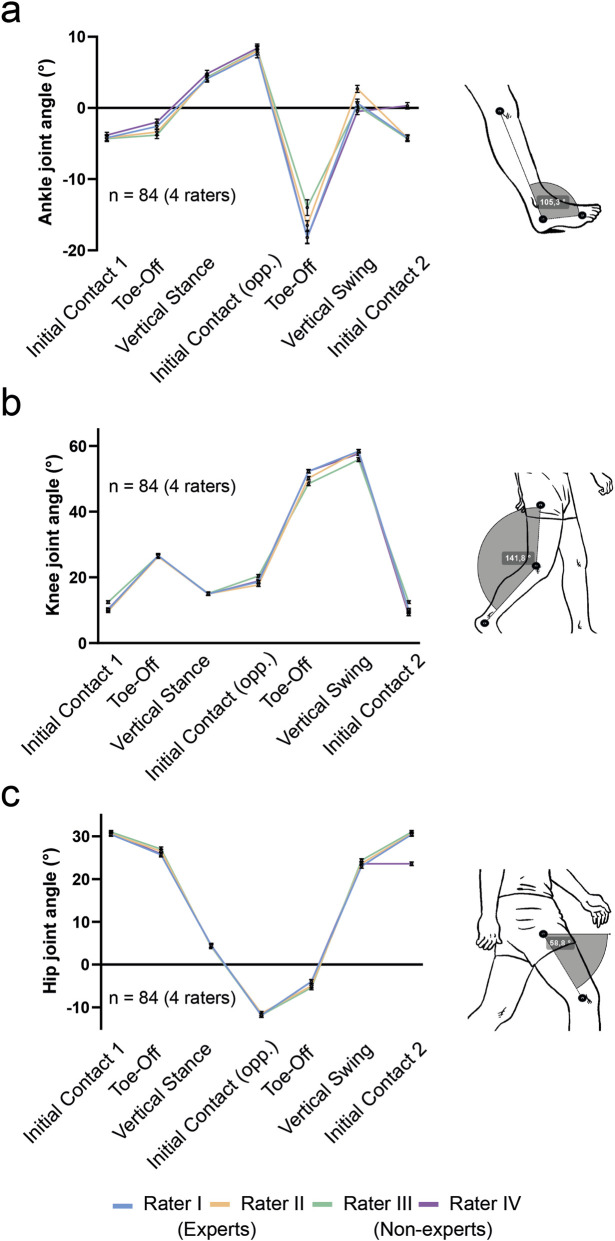
Lower-limb joint angle trajectories across gait events. **(a)** Mean ankle joint angle (degrees) across successive gait events, including initial contact, toe-off, vertical stance, opposite initial contact, opposite toe-off, vertical swing, and subsequent initial contact. Lines represent mean values obtained independently by four raters (Raters I–IV) across n = 84 participants. Negative values indicate plantarflexion and positive values indicate dorsiflexion. **(b)** Mean knee joint angle (degrees) across the same gait events, showing the characteristic increase in knee flexion during opposite toe-off and vertical swing phases, followed by extension at subsequent initial contact. **(c)** Mean hip joint angle (degrees) across gait events, illustrating the transition from hip flexion at initial contact to peak extension around opposite initial contact, and subsequent return to flexion during swing. Each colored line corresponds to one rater. Anatomical schematics illustrate joint angle definitions and sign conventions. Expert raters correspond to Raters I and II, whereas Raters III and IV correspond to non-expert raters following standardized protocol.

Next, the four raters analyzed lower-limb joint angle variables at the ankle, knee, and hip across the six gait events (initial contact, toe-off, vertical stance, opposite contact, opposite toe-off, and vertical swing). Descriptive inspection of joint angle trajectories revealed greater between-rater dispersion for ankle joint angles, particularly around toe-off and opposite toe-off events (Figure 3a). In contrast, a high degree of visual similarity across raters was observed for knee and hip angles throughout the gait cycle (Figures 3b,c).

In line with the descriptive findings, inter-examiner reliability for joint angle measurements was assessed using ICCs, with associated 95% CI, SEM, and MDC95 values presented in Table 4. For ankle joint angles, inter-examiner reliability ranged from moderate to excellent depending on the gait event. Initial contact showed good reliability (ICC = 0.76, 95% CI: 0.69–0.83), with a SEM of 0.95° and an MDC95 of 2.62°. Toe-off and vertical stance exhibited higher agreement (ICC = 0.79–0.86), with SEM values below 1.0° and MDC95 values between 2.25° and 2.74°. In contrast, opposite toe-off demonstrated markedly lower reliability (ICC = 0.45, 95% CI: 0.33–0.57), accompanied by a larger SEM (3.07°) and MDC95 (8.52°). Vertical swing showed moderate reliability (ICC = 0.63, 95% CI: 0.51–0.74), with intermediate measurement error (SEM = 1.52°, MDC95 = 4.22°). When stratified by examiner expertise, expert raters consistently achieved higher ICC values for ankle angles across all events (ICC range: 0.62–0.90) compared with non-expert raters (ICC range: 0.29–0.85), with experts also demonstrating smaller SEM and MDC95 values (Table 4).

**TABLE 4:** Inter-examiner reliability of lower-limb joint angle measurements across gait events for all examiners, experts, and non-experts.

Parameter	All				Experts				Non-experts			
	ICC	95% CI	SEM	MDC95	ICC	95% CI	SEM	MDC95	ICC	95% CI	SEM	MDC95
Ankle, initial contact	0.76	0.69–0.83	0.95	2.62	0.83	0.75–0.89	1.14	3.16	0.72	0.60–0.81	1.44	4.00
Ankle, toe-off	0.79	0.70–0.86	0.99	2.74	0.82	0.71–0.88	1.32	3.65	0.73	0.42–0.86	1.60	4.43
Ankle, vertical stance	0.86	0.82–0.90	0.81	2.25	0.90	0.85–0.94	0.97	2.67	0.85	0.77–0.90	1.23	3.42
Ankle, opposite contact	0.82	0.76–0.87	1.20	3.33	0.83	0.74–0.88	1.65	4.57	0.84	0.76–0.89	1.64	4.55
Ankle, opposite toe-off	0.45	0.33–0.57	3.07	8.52	0.62	0.47–0.74	3.16	8.76	0.29	0.08–0.47	5.46	15.14
Ankle, vertical swing	0.63	0.51–0.74	1.52	4.22	0.71	0.45–0.84	1.92	5.33	0.57	0.41–0.70	2.24	6.22
Knee, initial contact	0.73	0.58–0.83	1.10	3.04	0.83	0.75–0.89	1.22	3.39	0.63	0.24–0.80	1.76	4.88
Knee, toe-off	0.84	0.78–0.88	1.13	3.13	0.76	0.65–0.84	1.93	5.36	0.81	0.72–0.87	1.73	4.78
Knee, vertical stance	0.85	0.80–0.89	0.85	2.34	0.87	0.80–0.91	1.10	3.06	0.82	0.74–0.88	1.31	3.62
Knee, opposite contact	0.78	0.67–0.85	1.10	3.06	0.80	0.70–0.87	1.44	3.99	0.73	0.56–0.84	1.72	4.77
Knee, opposite toe-off	0.65	0.47–0.77	1.57	4.35	0.67	0.40–0.81	1.99	5.50	0.50	0.09–0.73	2.81	7.79
Knee, vertical swing	0.79	0.68–0.86	1.19	3.31	0.91	0.87–0.94	0.97	2.69	0.72	0.53–0.83	2.06	5.71
Hip, initial contact	0.86	0.80–0.90	0.72	1.98	0.78	0.68–0.85	1.26	3.50	0.92	0.88–0.95	0.75	2.09
Hip, toe-off	0.79	0.72–0.85	1.01	2.80	0.84	0.74–0.90	1.25	3.45	0.71	0.57–0.81	1.71	4.74
Hip, vertical stance	0.74	0.67–0.81	1.14	3.15	0.62	0.47–0.74	2.00	5.54	0.84	0.76–0.89	1.26	3.49
Hip, opposite contact	0.80	0.74–0.86	0.95	2.62	0.93	0.89–0.96	0.75	2.08	0.66	0.52–0.77	1.81	5.03
Hip, opposite toe-off	0.88	0.82–0.92	0.78	2.15	0.88	0.76–0.93	1.10	3.05	0.83	0.62–0.90	1.34	3.72
Hip, vertical swing	0.75	0.68–0.82	0.98	2.72	0.70	0.57–0.79	1.63	4.53	0.82	0.71–0.89	1.10	3.06

Abbreviations: ICC, intraclass correlation coefficient; 95% CI, 95% confidence interval; SEM, standard error of measurement; MDC95, minimal detectable change at the 95% confidence level.

Knee joint angle measurements showed moderate to good inter-examiner reliability across gait events. ICC values ranged from 0.65 to 0.85, with moderate reliability observed for opposite toe-off (ICC = 0.65) and initial contact (ICC = 0.73), and good reliability for toe-off, vertical stance, and vertical swing (ICC range: 0.79–0.85). Corresponding SEM values ranged from 1.10° to 1.57°, with MDC95 values between 2.34° and 4.35°. When stratified by examiner expertise, expert raters consistently demonstrated higher reliability than non-experts across all gait events, particularly for vertical stance and vertical swing, reaching ICC values up to 0.91. In contrast, non-expert raters showed lower ICC values (up to 0.82) and larger SEM and MDC95 values, indicating greater measurement error.

For hip joint angles, inter-examiner agreement was consistently high across gait events. The highest reliability was observed at opposite toe-off (ICC = 0.88, 95% CI: 0.82–0.92), accompanied by low measurement error (SEM = 0.78°, MDC95 = 2.15°). Initial contact also showed excellent reliability (ICC = 0.86, 95% CI: 0.80–0.90), with a SEM of 0.72° and an MDC95 of 1.98°. Vertical stance and vertical swing presented slightly lower, yet moderate-to-good, reliability (ICC = 0.74–0.75), with SEM values close to 1.0° and MDC95 ranging from 2.72° to 3.15°. Consistent with the patterns observed for ankle and knee joints, expert raters achieved higher ICC values and lower SEM and MDC95 estimates than non-expert raters across all hip gait events (Table 4).

### 3.4 Descriptive comparison of spatiotemporal parameters and joint kinematics with reference values

**FIGURE 4:**
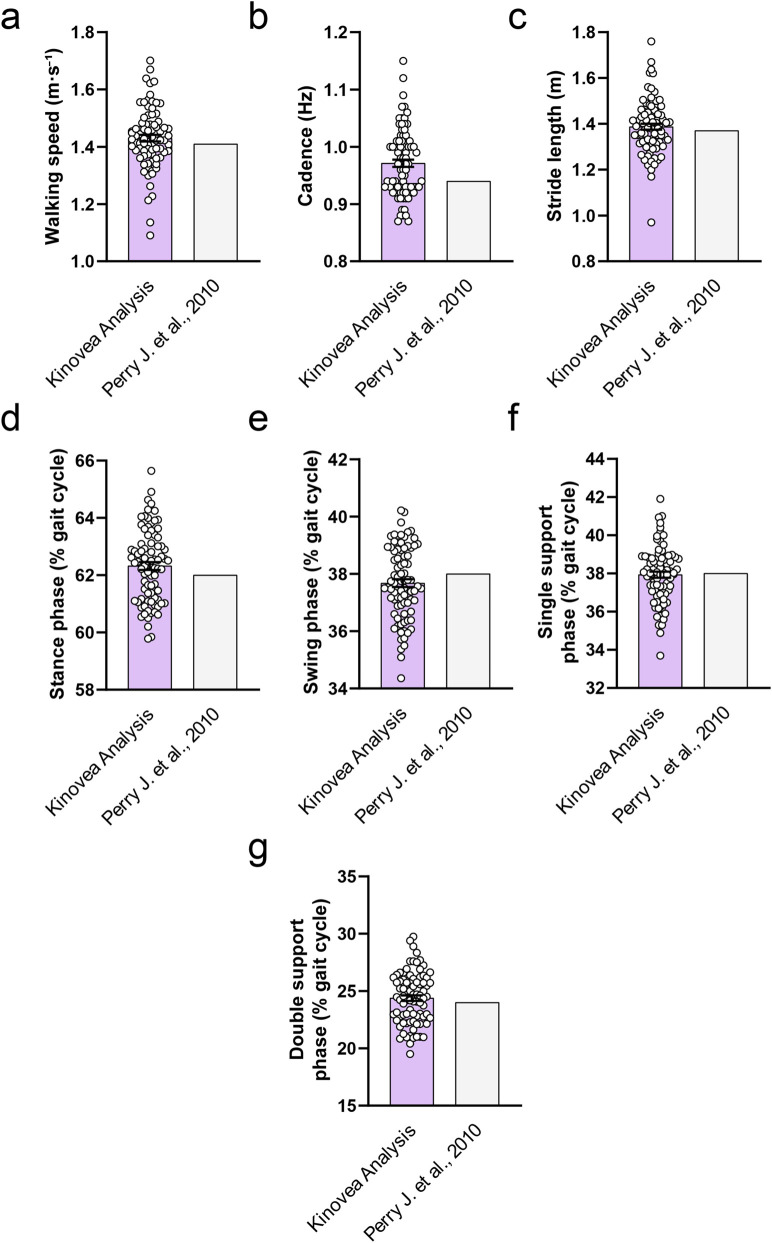
Descriptive comparison of Kinovea-derived spatiotemporal gait parameters with three-dimensional reference values. **(a)** Walking speed (m·s^-1^), **(b)** cadence (Hz), and **(c)** stride length (m) obtained from Kinovea-based 2D analysis in n = 84 healthy adults, contrasted with reference values reported by Perry and Burnfield (2010). **(d)** Stance phase, **(e)** swing phase, **(f)** single-support phase, and **(g)** double-support phase expressed as percentages of the gait cycle and displayed in the same manner. Across panels, individual points represent single-participant measurements, coloured bars indicate Kinovea-derived mean values, and grey bars denote published three-dimensional values. This descriptive comparison provides contextualization of Kinovea-derived spatiotemporal parameters relative to established gait data.

After completion of the inter-examiner reliability assessment, spatiotemporal gait parameters obtained from the 84 healthy participants using Kinovea were compared with values derived from three-dimensional motion capture systems in healthy populations (Perry and Burnfield, 2010) (Figure 4). These comparisons are purely descriptive and are not intended to represent a validation against three-dimensional motion capture systems, as no direct concurrent measurements were performed within the same experimental framework. Walking speed showed a mean value of 1.39 ± 0.12 m s^-1^, with a 95% CI of 1.36–1.41 m s^-1^, closely matching the reference value of 1.41 m s^-1^ (Figure 4a). Cadence was 0.97 ± 0.06 Hz (95% CI: 0.96–0.98 Hz), slightly higher but still consistent with the reference value of 0.94 Hz (Figure 4b). Similarly, stride length averaged 1.43 ± 0.11 m (95% CI: 1.41–1.45 m), comparable to the reference value of 1.37 m (Figure 4c).

Regarding temporal gait phases, the stance phase accounted for 62.3% ± 1.3% of the gait cycle (95% CI: 62.1%–62.6%), in close agreement with the reference value of 62% (Figure 4d). The swing phase represented 37.7% ± 1.3% of the gait cycle (95% CI: 37.4%–38.0%), consistent with the reference value of 38% (Figure 4e). Likewise, single-support phase duration averaged 37.9% ± 1.5% of the gait cycle (95% CI: 37.6%–38.3%), aligning well with the reference value of 38% (Figure 4f), while double-support phase duration showed a mean value of 24.4% ± 2.3% (95% CI: 23.9%–24.9%), comparable to the reference value of 24% (Figure 4g).

**FIGURE 5:**
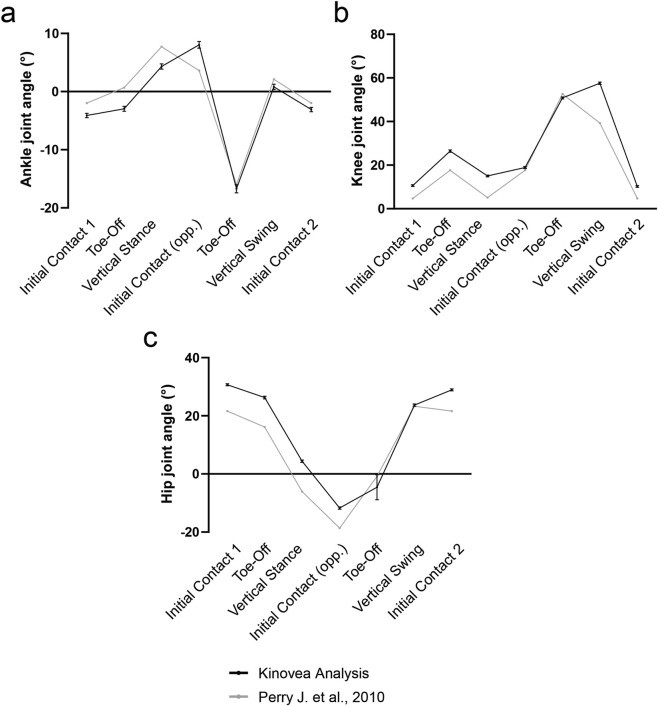
Descriptive comparison of lower-limb joint angle trajectories across gait events between Kinovea analysis and reference data. **(a)** Ankle joint angle, **(b)** knee joint angle, and **(c)** hip joint angle trajectories extracted at different gait events (initial contact, toe-off, vertical stance, opposite initial contact, opposite toe-off, vertical swing, and subsequent initial contact). Black lines represent mean joint angles derived from Kinovea-based 2D analysis in n = 84 healthy adults, whereas grey lines denote corresponding reference trajectories reported by Perry and Burnfield (2010). Angles are expressed in degrees and plotted according to a consistent sign convention across joints. This descriptive comparison contextualizes Kinovea-derived kinematic profiles relative to three-dimensional gait reference patterns.

Additionally, joint kinematic data obtained using Kinovea were descriptively contrasted across gait events (Figure 5). Ankle joint angles derived from Kinovea exhibited the expected directional changes across the gait cycle (Figure 5a). At initial contact, the ankle angle was −4.1° ± 3.5° (95% CI: −4.88 to −3.35), compared with −2° reported by Perry and Burnfield (2010). At toe-off, the ankle angle was −3.4° ± 4.0° (95% CI: −3.83 to −3.10), whereas Perry and Burnfield (2010) reported 0.7°. During vertical stance, ankle dorsiflexion increased to 4.3° ± 4.2° (95% CI: 3.41–5.22), compared with 7.7°. At opposite initial contact, the ankle angle reached 8.1° ± 5.3° (95% CI: 6.90–9.20), while the reference value was 3.6°. At opposite toe-off, the largest plantarflexion was observed (−16.7° ± 6.3°, 95% CI: −18.08 to −15.34), which was close in magnitude to the reference value (−16°). During vertical swing, the ankle angle returned towards neutral (0.8° ± 4.3°, 95% CI: −0.11–1.75), compared with 2.1°. Values at the subsequent initial contact mirrored those observed at the beginning of the gait cycle.

Knee angles showed marked changes across events (Figure 5b). At initial contact, knee flexion was 10.6° ± 3.8° (95% CI: 9.82–11.46), compared with 4.7° in Perry and Burnfield (2010). At toe-off, knee flexion increased to 26.5° ± 5.2° (95% CI: 25.32–27.59), versus 17.6°, and decreased during vertical stance (15.0° ± 4.1°, 95% CI: 14.12–15.89), compared with 5.1°. At opposite initial contact, knee flexion was 18.9° ± 4.3° (95% CI: 17.98–19.85), close to the reference value (17.6°). At opposite toe-off, knee flexion reached 50.8° ± 4.5° (95% CI: 49.84–51.80), similar in magnitude to the reference value (52.6°). During vertical swing, knee flexion was 57.6° ± 4.8° (95% CI: 56.53–58.59), higher than the reference value (39.3°). At the subsequent initial contact, knee flexion returned to 10.6° ± 3.8° (95% CI: 9.82–11.46), compared with 4.7°.

Hip angles derived from Kinovea followed a progression from flexion to extension and back to flexion over the gait cycle (Figure 5c). At initial contact, hip flexion was 30.7° ± 3.6° (95% CI: 29.92–31.46), compared with 21.6° in Perry and Burnfield (2010), and remained flexed at toe-off (26.3° ± 4.1°, 95% CI: 25.38 to 27.14° vs. 16.1°). During vertical stance, the hip angle approached neutral (4.4° ± 4.0°, 95% CI: 3.48–5.23), whereas the reference value was −6.1°. Peak extension occurred at opposite initial contact (−11.7° ± 3.9°, 95% CI: −12.57 to −10.86 compared with −18.6°). At opposite toe-off, hip angle was −4.6° ± 4.3° (95% CI: −5.50 to −3.64 vs. −0.8°). During vertical swing, hip flexion increased to 23.7° ± 3.6° (95% CI: 22.89–24.45), comparable to the reference value (23.3°). Hip flexion at the subsequent initial contact was consistent with values observed at the start of the gait cycle (30.7° ± 3.6°, 95% CI: 29.92 to 31.46° vs. 21.6°).

Overall, these comparisons are presented for contextual purposes only and should be interpreted as descriptive benchmarks to assess the plausibility and external coherence of the measurements, rather than as evidence of validity against three-dimensional motion capture systems.

### 3.5 Agreement analysis between examiners

Given the large number of spatiotemporal and kinematic variables analyzed (Tables 3, 4), agreement between expert and non-expert examiners was not evaluated for all parameters. Instead, a subset of representative outcomes was selected for analysis. Variables were chosen based on their widespread use in gait analysis, their clinical and biomechanical relevance, and their ability to illustrate different levels of inter-examiner agreement across both spatiotemporal and kinematic domains. Specifically, walking speed, cadence, and stride length were selected as canonical spatiotemporal descriptors, while ankle, knee, and hip joint angles at key gait events were included to represent lower-limb kinematics with varying reliability profiles.

**FIGURE 6:**
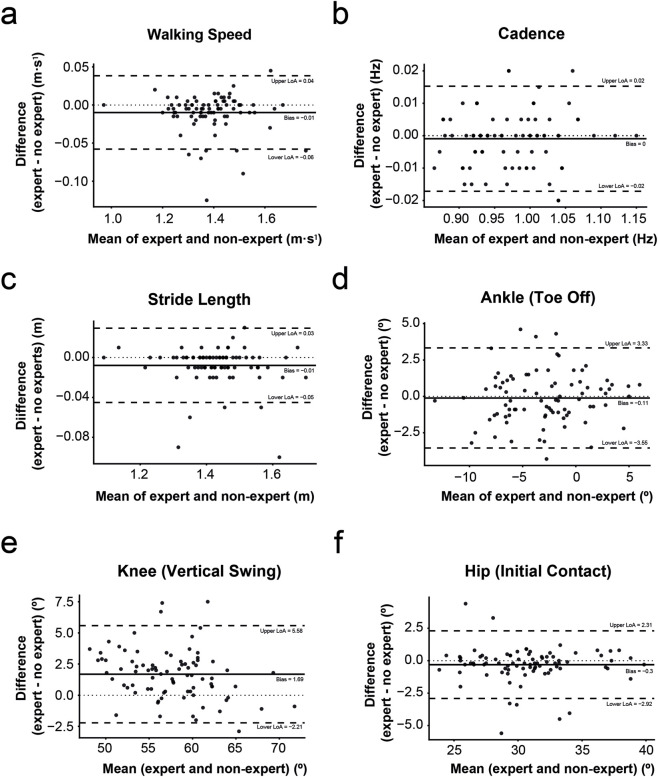
Bland–Altman agreement analysis between expert and non-expert examiners for spatiotemporal and kinematic gait parameters. **(a)** Walking speed, **(b)** cadence, and **(c)** stride length. **(d)** Ankle joint angle at toe-off, **(e)** knee joint angle during vertical swing, and **(f)** hip joint angle at initial contact. For each panel, the difference between expert and non-expert measurements (expert minus non-expert) is plotted against their mean value for n = 84 participants. Solid horizontal lines indicate mean bias, and dashed lines denote the 95% limits of agreement.

Bland–Altman plots were generated for both spatiotemporal gait parameters and lower-limb joint kinematics (Figure 6). For each variable, the mean of expert and non-expert measurements was plotted against their difference (expert minus non-expert), allowing the estimation of systematic bias and 95% limits of agreement (LoA). Spatiotemporal parameters, including walking speed, cadence, and stride length, showed negligible systematic bias (≤0.01 in absolute terms) and narrow 95% limits of agreement, indicating good agreement between expert and non-expert examiners across the full range of measured values (Figures 6a–c). In contrast, kinematic variables exhibited wider limits of agreement, despite relatively small mean biases, particularly for ankle angle at toe-off (LoA: −3.55° to 3.33°) and knee angle during vertical swing (LoA: −2.21°–5.58°), reflecting greater inter-examiner variability in joint angle estimation (Figures 6d–f).

### 3.6 Sex-stratified descriptive gait parameter dataset derived from kinovea-based 2D video analysis

To facilitate the implementation and interpretation of Kinovea-derived 2D gait parameters, a sex-stratified descriptive reference table of spatiotemporal and joint kinematic variables was generated from the healthy cohort (n = 84; females n = 34, males n = 50). For each parameter, mean values, standard deviations, and 95% confidence intervals are presented separately for the total sample and by sex (Table 5). Given the influence of anthropometric characteristics (Table 1), stratification by sex provides a contextual framework for interpreting stride length, cadence, walking speed, and lower-limb joint angles. These sex-stratified data are purely descriptive. No statistical comparisons between females and males were performed, and apparent sex-related differences should not be interpreted as inferential evidence of true kinematic differences. In particular, differences in angular variables may reflect methodological factors inherent to 2D analysis, including camera alignment, segment projection, or marker placement variability.

**TABLE 5:** Sex-stratified descriptive statistics of spatiotemporal gait parameters and lower-limb joint angles derived from Kinovea-based 2D analysis in healthy adults.

Parameter	All (n = 84)		Female (n = 34)		Male (n = 50)	
	Mean ± SD	95% CI	Mean ± SD	95% CI	Mean ± SD	95% CI
Stride length (m)	1.43 ± 0.11	1.41 to 1.45	1.41 ± 0.15	1.36 to 1.46	1.45 ± 0.10	1.42 to 1.48
Walking speed (m·s^-1^)	1.39 ± 0.12	1.36 to 1.41	1.40 ± 0.16	1.34 to 1.45	1.38 ± 0.10	1.36 to 1.41
Cadence (Hz)	0.97 ± 0.06	0.96 to 0.98	0.99 ± 0.06	0.97 to 1.01	0.96 ± 0.05	0.94 to 0.97
Stance phase (% gait cycle)	62.32 ± 1.26	62.05 to 62.59	62.04 ± 1.34	61.57 to 62.50	62.52 ± 1.17	62.19 to 62.86
Swing phase (% gait cycle)	37.68 ± 1.26	37.41 to 37.95	37.96 ± 1.34	37.50 to 38.43	37.48 ± 1.17	37.15 to 37.81
Single-support phase (% gait cycle)	37.93 ± 1.47	37.61 to 38.25	38.24 ± 1.17	37.84 to 38.65	37.72 ± 1.62	37.26 to 38.18
Double-support phase (% gait cycle)	24.39 ± 2.25	23.90 to 24.88	23.79 ± 2.13	23.05 to 24.54	24.80 ± 2.25	24.16 to 25.44
Ankle, initial contact (°)	−4.12 ± 3.52	−4.88 to −3.35	−2.45 ± 2.87	−3.45 to −1.45	−5.25 ± 3.50	−6.25 to −4.26
Ankle, toe-off (°)	−3.35 ± 3.98	−3.83 to −2.10	−2.39 ± 4.09	−3.82 to −0.96	−3.36 ± 3.89	−4.46 to −2.25
Ankle, vertical stance (°)	4.31 ± 4.19	3.41 to 5.22	5.67 ± 3.82	4.34 to 7.01	3.38 ± 4.21	2.19 to 4.58
Ankle, opposite contact (°)	8.05 ± 5.29	6.90 to 9.20	9.14 ± 5.13	7.35 to 10.93	7.30 ± 5.32	5.79 to 8.81
Ankle, opposite toe-off (°)	−16.71 ± 6.32	−18.08 to −15.34	−17.40 ± 7.13	−19.89 to −14.92	−16.24 ± 5.74	−17.87 to −14.61
Ankle, vertical swing (°)	0.82 ± 4.29	−0.11 to 1.75	1.13 ± 4.26	−0.36 to 2.62	0.60 ± 4.35	−0.63 to 1.84
Knee, initial contact (°)	10.64 ± 3.79	9.82 to 11.46	11.76 ± 3.58	10.51 to 13.01	9.89 ± 3.77	8.82 to 10.96
Knee, toe-off (°)	26.45 ± 5.23	25.32 to 27.59	27.01 ± 6.32	24.80 to 29.22	26.08 ± 4.37	24.84 to 27.32
Knee, vertical stance (°)	15.01 ± 4.08	14.12 to 15.89	15.66 ± 4.22	14.19 to 17.13	14.57 ± 3.96	13.44 to 15.69
Knee, opposite contact (°)	18.92 ± 4.30	17.98 to 19.85	20.05 ± 5.41	18.17 to 21.94	18.15 ± 3.19	17.24 to 19.05
Knee, opposite toe-off (°)	50.82 ± 4.53	49.84 to 51.80	50.42 ± 5.64	48.45 to 52.39	51.08 ± 3.63	50.05 to 52.12
Knee, vertical swing (°)	57.56 ± 4.76	56.53 to 58.59	58.41 ± 5.22	56.58 to 60.23	56.98 ± 4.37	55.74 to 58.22
Hip, initial contact (°)	30.69 ± 3.57	29.92 to 31.46	32.16 ± 3.67	30.88 to 33.44	29.69 ± 3.16	28.79 to 30.59
Hip, toe-off (°)	26.26 ± 4.07	25.38 to 27.14	27.07 ± 3.99	25.68 to 28.46	25.70 ± 4.07	24.55 to 26.86
Hip, vertical stance (°)	4.36 ± 4.04	3.48 to 5.23	4.84 ± 4.28	3.35 to 6.33	4.03 ± 3.88	2.92 to 5.13
Hip, opposite contact (°)	−11.72 ± 3.94	−12.57 to −10.86	−11.79 ± 4.92	−13.50 to −10.07	−11.67 ± 3.16	−12.57 to −10.77
Hip, opposite toe-off (°)	−4.57 ± 4.29	−5.50 to −3.64	−5.93 ± 5.28	−7.77 to −4.09	−3.65 ± 3.21	−4.56 to −2.74
Hip, vertical swing (°)	23.67 ± 3.58	22.89 to 24.45	24.83 ± 3.53	23.60 to 26.07	22.88 ± 3.44	21.90 to 23.86

Abbreviations: CI, confidence interval; SD, standard deviation; n, sample size; m, meters; m·s^-1^, meters per second; Hz, hertz; %, percentage of gait cycle; °, degrees.

## 4 Discussion

Gait analysis represents an important tool in both research and clinical practice, providing quantitative information that supports diagnostic reasoning, treatment planning, and outcome monitoring (Chambers and Sutherland, 2002; Perry and Burnfield, 2010; Papagiannis et al., 2018; Kour et al., 2021). Despite its well-established value, its implementation in routine clinical settings remains largely confined to specialized centers. This limitation is often driven by the perceived technical complexity of gait assessment and the assumption that accurate evaluation requires costly laboratory-based technologies (Spallone et al., 2025). Although gait can be assessed through approaches ranging from visual inspection to advanced biomechanical instrumentation, optoelectronic motion capture systems combined with force platforms remain the reference standard in many research contexts (Hulleck et al., 2022). The prominence of these technologies may reinforce the notion that precise gait analysis is intrinsically dependent on high-end laboratory infrastructure, thereby restricting broader clinical adoption.

In the present study, we examined the inter-rater reliability of a two-dimensional video-based methodology for gait analysis using a recording setup and subsequent analysis with the open-source software Kinovea. This platform has been explored across diverse research contexts, where it has shown acceptable levels of validity and reliability compared with established motion capture systems (Fernández-González et al., 2020; Iijima et al., 2023; Menychtas et al., 2023; Faj et al., 2025; Vicente-Pina et al., 2025). Previous investigations have reported small discrepancies in joint angle measurements during dynamic tasks related to optoelectronic systems, strong agreement in spatiotemporal variables such as flight time and jump height when compared to infrared platforms, and accurate distance and angular parameters quantification under controlled conditions (Balsalobre-Fernández et al., 2014; Puig-Diví et al., 2019; Fernández-González et al., 2020). Despite this growing body of validation studies, important methodological aspects remain insufficiently addressed. Inter-rater reliability—and whether examiner expertise influences measurement consistency—have not been systematically evaluated in powered samples.

Building on this foundation, our findings indicate that a 2D video-based gait analysis approach provides reliable measurements for global spatiotemporal parameters, largely independent of examiner expertise (Table 3). Walking speed, cadence, and stride length showed excellent inter-examiner agreement with minimal measurement error, and similarly high ICC values were observed among non-expert raters. In contrast, temporal gait phase variables and selected joint kinematics—particularly distal segments and event-dependent ankle angles—demonstrated lower reliability, reflecting intrinsic constraints of sagittal plane 2D analysis that are only partially mitigated by training (Table 4). Despite these limitations, mean values remained biomechanically coherent and broadly consistent with established 3D reference data (Figure 4). Collectively, these results support the cautious use of 2D gait analysis for selected variables in healthy young adults, particularly global spatiotemporal parameters.

The heterogeneity observed in inter-rater reliability across the analyzed parameters likely reflects intrinsic differences in their computational structure and susceptibility to measurement error. Global descriptors, such as walking speed, are derived from spatiotemporal markers that are relatively insensitive to minor frame-by-frame discrepancies during event detection. Consequently, agreement between experienced and inexperienced examiners remains high, as minor temporal misalignments exert negligible influence on the final estimate. However, prior methodological investigations have emphasized the vulnerability of event-dependent parameters to limitations in frame resolution and subjective visual interpretation in 2D recordings (Iijima et al., 2023; Menychtas et al., 2023). In line with these reports, our findings indicate that temporal gait phase variables exhibit greater heterogeneity, primarily because they rely on the precise identification of discrete gait events within a constrained temporal resolution. Even minimal deviations in marking toe-off or contralateral toe-off can propagate through percentage-based calculations, thereby amplifying variability. Although the mean values of these parameters approximated those described in 3D datasets, their reduced reliability tempers their suitability for longitudinal monitoring or multi-examiner comparisons.

Variability in joint kinematics has been documented in prior two-dimensional gait analyses (Fernández-González et al., 2020; Faj et al., 2025), underscoring the methodological constraints inherent to planar recordings. Consistent with these observations, our angular parameters exhibited a clear proximal-to-distal gradient: hip angles demonstrated the highest consistency, knee angles showed moderate agreement, and ankle angles presented the greatest variability. Beyond the sensitivity to frame selection discussed, angular measurements are influenced by subtle deviations in marker placement and by the exact pixels chosen during digitization. These sources of error are further compounded by camera perspective and out-of-plane motion. In 2D analyses, such factors likely introduce projection errors that disproportionately affect distal segments, where smaller anatomical lever arms amplify minor positional deviations into larger angular discrepancies. Together, these results indicate that not all gait metrics are equally transferable across methodological scales, and that the utility of 2D approaches depends on careful parameter selection.

Previous research in biomechanical assessment contexts has shown that examiner experience does not necessarily influence inter-rater reliability when standardized acquisition protocols are applied. In three-dimensional gait analysis, comparable reliability has been reported between experienced and novice testers following basic training (Leigh et al., 2014). Our results showed that examiner expertise improved measurement consistency, particularly for joint kinematics, where non-expert raters consistently achieved lower measurement error. However, expertise did not fully overcome the intrinsic methodological constraints affecting distal joints and event-dependent variables. Importantly, global spatiotemporal parameters remained highly reliable even among non-expert raters, reinforcing the robustness of these outcomes.

Despite variability in certain kinematic measures, mean spatiotemporal and angular values closely approximated established three-dimensional normative data. Observed angular offsets were systematic rather than random. This aspect has been reported in the literature (Menychtas et al., 2023) and is largely attributed to methodological factors inherent to two-dimensional analysis. Specifically, sagittal-plane projection compresses three-dimensional movement into a single plane, which may distort true joint angles, particularly in the presence of out-of-plane motion. In addition, small deviations in camera alignment relative to the participant’s plane of progression, as well as differences in segment definition and marker placement compared to multi-marker 3D models, may introduce consistent angular offsets. Together, these factors can lead to systematic overestimation or underestimation of joint angles without necessarily increasing measurement variability across evaluators. These differences should not be interpreted as measurement error in the classical sense, but rather as method-specific offsets inherent to two-dimensional kinematic estimation. In this context, the present findings support the external coherence of the methodology while acknowledging its structural limitations, and should be interpreted within the scope of inter-rater reliability rather than absolute agreement with three-dimensional motion capture systems.

A relevant methodological consideration is that analyses were performed on a single gait cycle per participant. In clinical gait analysis, averaging several consecutive cycles is generally preferable when the aim is to characterize an individual’s habitual gait pattern, because gait exhibits inherent stride-to-stride variability even in healthy adults. However, in the present study, the primary objective was not to estimate intra-individual gait variability, but to determine whether different examiners could obtain consistent measurements from the same standardized video sequence using a 2D workflow. Selecting the gait cycle that was best centered and most orthogonal to the camera reduced the influence of perspective distortion and marker visibility issues, which are particularly relevant in sagittal-plane 2D analysis. Therefore, this approach improved standardization for inter-rater reliability assessment, but at the cost of limiting the representativeness of the extracted values as estimates of each participant’s habitual gait.

From a pragmatic standpoint, this methodology may represent a feasible option for quantitative gait assessment in controlled settings when laboratory-based systems are unavailable or impractical. Its strengths lie in global spatiotemporal analysis and selected proximal kinematic outcomes, suggesting potential utility in assessment contexts, provided that variable selection and methodological limitations are carefully considered. However, it should not be considered a surrogate for comprehensive 3D motion analysis and these findings must be interpreted alongside emerging motion analysis approaches, including markerless computer vision–based systems and wearable sensors. Markerless systems offer the advantage of fully automated data extraction and improved ecological validity, enabling movement assessment in unconstrained environments. However, current validation studies suggest that their accuracy remains dependent on algorithm performance, camera configuration, and task complexity, particularly in scenarios involving occlusions or out-of-plane motion. Similarly, wearable inertial measurement units (IMUs) provide continuous, real-world gait monitoring but are subject to limitations related to sensor drift, calibration procedures, and model-based estimation of joint kinematics. Within this evolving technological landscape, the present marker-assisted two-dimensional approach should be understood as a controlled intermediate workflow between observational gait analysis and fully automated markerless systems. In the context of the present study, this approach enables improved anatomical landmark identification and measurement transparency under standardized conditions, rather than prioritizing ecological validity. This positioning may be particularly relevant for controlled assessment contexts, although its performance in clinical populations requires further investigation.

### 4.1 Limitations

First, the present findings were derived from a cohort of healthy young adults, and caution is warranted when extrapolating this methodology to individuals with altered gait patterns, where movement variability and compensatory strategies may amplify measurement error. Reliability estimates may therefore differ in older adults, patients with neurological or musculoskeletal disorders, or individuals with more variable or compensatory gait patterns.

Second, the analysis was based on a single gait cycle per participant. This approach was chosen to ensure that all raters analyzed the same optimally visible and geometrically appropriate cycle, thereby reducing perspective-related error and improving standardization for inter-rater reliability assessment. However, single-cycle analysis does not capture stride-to-stride variability and may provide a less robust estimate of each participant’s habitual gait pattern than analyses based on multiple averaged cycles.

Third, reliability was evaluated exclusively for the data editing and reduction process, whereas the reproducibility of laboratory setup, camera positioning, and marker placement was not formally tested and may introduce additional variability in real-world implementations. In addition, the present study did not include a direct concurrent comparison with a three-dimensional motion capture system; therefore, the descriptive comparisons with literature-based reference values should not be interpreted as evidence of validity, but rather as contextual benchmarks of measurement plausibility.

Fourth, the analysis was restricted to sagittal plane kinematics of the hip, knee, and ankle, and therefore does not capture frontal or transverse plane mechanics, which may be clinically relevant in certain conditions. In addition, the relatively short camera-to-walkway distance (3 m) chosen to enhance clinical feasibility may have increased susceptibility to perspective distortion, contributing to systematic angular offsets inherent to two-dimensional projection.

Fifth, intra-rater reliability was not assessed. The present study was designed primarily to evaluate inter-rater reliability across examiners with different levels of expertise, as this was considered the most relevant source of variability for multi-examiner clinical and educational implementation. However, the absence of repeated measurements by the same examiner prevents us from determining whether individual raters would reproduce their own measurements consistently over time. This is particularly relevant for manually identified gait events and event-dependent joint angles, where subjective frame selection may influence measurement stability. Future studies should include both intra-rater and inter-rater reliability analyses, ideally using repeated analyses separated by an adequate washout period and including multiple gait cycles per participant.

Finally, occasional marker occlusion—particularly at the greater trochanter due to upper limb swing—may have introduced minor uncertainty in specific frames, although this reflects a practical constraint commonly encountered in video-based gait analysis. Together, these factors should be considered when interpreting the generalizability and scope of the proposed methodology.

## 5 Conclusion

In healthy young adults, this study shows that a two-dimensional video-based system using Kinovea can provide reliable measurements for global spatiotemporal gait parameters under standardized conditions, with strong inter-examiner agreement even among non-expert raters. Joint kinematic reliability was joint- and event-dependent, showing greater consistency for proximal segments and selected gait events, whereas distal joint angles and temporal gait phase variables were more sensitive to examiner-related and methodological constraints inherent to sagittal-plane 2D analysis. Descriptive comparisons with three-dimensional reference data suggest biomechanical coherence but should not be interpreted as formal validation against 3D motion capture. Overall, these findings support the cautious use of this methodology for selected gait variables in healthy young adults. Further studies are needed to determine whether similar reliability estimates apply to older adults and clinical populations with gait pathology, where movement variability may be higher.

## Data Availability

The raw data supporting the conclusions of this article will be made available by the authors, without undue reservation.
