# Supplementary material for: Accessible 2D video-based system for gait kinematic analysis: an inter-rater reliability study
Source: Front Bioeng Biotechnol. 2026 Jul 20;14:1815411. doi: 10.3389/fbioe.2026.1815411 (PMC13429743; doi:10.3389/fbioe.2026.1815411)
Supplement: Supplementary file 1 [file Table1.docx]

**Supplementary Material 1. Step-by-step workflow for gait video analysis and event identification.**

1. Open kinovea’s software (available: <https://kinovea.org/>)
2. Open the video file you want to edit
3. Improve visibility: “View” + “Full screen or F11”
4. Calibration:
   1. Select “Line” tool to draw a line between the 2 markers located on the floor
   2. Select “Move” tool and click the right mouse button on the drawn line
   3. Select “Calibration” to configure the distance with the separation between markers
5. Delete the key image created automatically
6. Select the most centered gait cycle (GC)
7. Create the 7 events with "Add a key image" tool
8. Calculate stride length:
   1. Select “Line” tool to draw a line between the calcaneal markers of the 1st and 7th events. To achieve this, select “Move” tool and click the right mouse button on the drawn line and select "Visibility” and “Always visible”
9. Three stopwatches are set up with "Stopwatch" tool in an area that does not obstruct the video:
   1. The first one is for the GC and starts and stops between events 1 and 7
   2. The second one is for the Single Support and starts and stops between events 2 and 4
   3. The third one is for the Swing and starts and stops between events 5 and 7
10. Calculate the ankle, knee, and hip angles for each event with "Angle" tool
11. Close and save de editions with “Save annotations” tool

Some tips during the editing of this process:

- Use the right and left arrow keys on your keyboard to move forward or backward frame by frame
- Zoom in on the video using the CTRL key and mouse scroll wheel
- If the value of a distance is not displayed, select “Move” tool and click the right mouse button on the drawn line and select "Label” and “Length”
- After closing and saving the edit, the program will automatically create a file with the same name as the video and the .kva extension in the same directory where the video file is located. To reopen the edited file, only the video file should be opened, but the file with the .kva extension must be in the same directory.
