# Supplementary material for: Accessible 2D video-based system for gait kinematic analysis: an inter-rater reliability study
Source: Front Bioeng Biotechnol. 2026 Jul 20;14:1815411. doi: 10.3389/fbioe.2026.1815411 (PMC13429743; doi:10.3389/fbioe.2026.1815411)
Supplement: Supplementary file 2 [file Table2.docx]

**Supplementary Material 2. Operational criteria for gait event identification.**

| Event | Abbreviation | Operational Definition |
| --- | --- | --- |
| First Initial Contact | IC1 | First heel contact with the ground. When frame selection generated uncertainty (i.e., between a frame where the heel had not yet contacted and one where contact was evident), the frame showing visible ground contact was selected. |
| Toe-Off Opposite | TOop | Toe-off of the contralateral limb. When analyzing a Right Gait Cycle (Right-GC), this event corresponds to the left foot. |
| Vertical Stance | VSt | Mid-stance instant defined when the tips of both feet were aligned horizontally at the same vertical level. |
| Initial Contact Opposite | ICop | Heel strike of the contralateral limb. |
| Toe-Off | TO | Toe-off of the ipsilateral limb (end of stance phase). |
| Vertical Swing | VSw | Mid-swing instant defined when the tips of both feet were aligned horizontally at the same vertical level during the swing phase. |
| Second Initial Contact | IC2 | Second heel contact of the ipsilateral limb, marking the end of the analyzed gait cycle. |
