# Supplementary material for: Accessible 2D video-based system for gait kinematic analysis: an inter-rater reliability study
Source: Front Bioeng Biotechnol. 2026 Jul 20;14:1815411. doi: 10.3389/fbioe.2026.1815411 (PMC13429743; doi:10.3389/fbioe.2026.1815411)
Supplement: Supplementary file 3 [file Table3.docx]

**Supplementary Material 3. Formulae and computational procedures for the calculation of spatiotemporal gait parameters and joint angle correction.**

| Parameter | Abbreviation | Unit | Computational Formula | Operational Definition |  | Joint |  | Abbreviation | Anatomical Correction Formula |
| --- | --- | --- | --- | --- | --- | --- | --- | --- | --- |
| Stride Length | STRL | m | $STRL=\left\vert x_{IC2}-x_{IC1} \right\vert$ | Linear displacement between first and second ipsilateral initial contact |  | Ankle |  | ANK | $\theta_{ANK}={90}^{\circ}-\theta_{ANK}^{raw}$ |
| Velocity | VEL | m/s | $VEL=\frac{STRL}{T_{GC}}$ | Mean forward velocity across one gait cycle |  | Knee |  | KNEE | $\theta_{KNEE}={180}^{\circ}-\theta_{KNEE}^{raw}$ |
| Cadence | CAD | s^−1^ | $CAD=\frac{1}{T_{GC}}$ | Number of gait cycles per second |  | Hip |  | HIP | $\theta_{HIP}={90}^{\circ}-\theta_{HIP}^{raw}$ |
| Stance Phase | St | % GC | $St=\frac{T_{St}}{T_{GC}}\times100$ | Percentage of gait cycle spent in stance phase |  |  |  |  |  |
| Swing Phase | Sw | % GC | $Sw=\frac{T_{Sw}}{T_{GC}}\times100$ | Percentage of gait cycle spent in swing phase |  |  |  |  |  |
| Single Support | SSup | % GC | $SSup=\frac{T_{SSup}}{T_{GC}}\times100$ | Percentage of gait cycle spent on monopodal support |  |  |  |  |  |
| Double Support | DSup | % GC | $DSup=\frac{T_{DSup}}{T_{GC}}\times100$ | Percentage of gait cycle spent on bipodal support |  |  |  |  |  |

Temporal variables were defined as follows: $T_{GC}=t_{IC2}-t_{IC1}$ ; $T_{Sw}=t_{IC2}-t_{TO}$ ; $T_{St}=T_{GC}-T_{Sw}$ ; $T_{SSup}=t_{ICop}-t_{TOop}$ ; $T_{DSup}=T_{St}-T_{SSup}$. All percentage-based parameters were normalized to $T_{GC}$.
